# A note on utilising binary features as ligand descriptors

**DOI:** 10.1186/s13321-015-0105-3

**Published:** 2015-12-01

**Authors:** Hamse Y. Mussa, John B. O. Mitchell, Robert C. Glen

**Affiliations:** Centre for Molecular Informatics, Department of Chemistry, Cambridge University, Lensfield Road, Cambridge, CB2 1EW UK; EaStCHEM School of Chemistry and Biomedical Sciences Research Complex, University of St Andrews, North Haugh, St Andrews, KY16 9ST St Andrews, Scotland

**Keywords:** Binary descriptors, Ligand chemical structure, Linear relationship, Bernoulli distribution

## Abstract

It is common in cheminformatics to represent the properties of a ligand as a string of 1’s and 0’s, with the intention of elucidating, *inter alia*, the relationship between the chemical structure of a ligand and its bioactivity. In this commentary we note that, where relevant but non-redundant features are binary, they inevitably lead to a classifier capable of capturing only a linear relationship between structural features and activity. If, instead, we were to use relevant but non-redundant real-valued features, the resulting predictive model would be capable of describing a non-linear structure-activity relationship. Hence, we suggest that real-valued features, where available, are to be preferred in this scenario.

## Background

One of the major goals of cheminformatics is to predict the relationship between a ligand’s chemical structure and its bioactivity [1]. If this relationship is captured correctly, then (among other goals) designing the right drug for each disease would become an easier task [1, 2]. Unfortunately, the structure-activity relationship can often be intricate and arcane, and in particular non-linear.

To devise an adequate model describing this relationship, the cheminformaticist typically follows a standard approach; starting with a large number of ligand attributes or features considered important for representing the underlying characteristics of the ligand, and relevant to its bioactivity. Then, through feature selection techniques, one selects the ligand attributes deemed to have statistically minimum interdependence among themselves (given the ligand bioactivity), while also showing strong association with the ligand bioactivity [3–5]. With this step, one strives for a set of relevant but non-redundant ligand features [4, 5]: “relevant” in the sense that there is a strong association between the selected features and the bioactivity, and “non-redundant” in the sense that these features are conditionally independent given the bioactivity. (Irrelevant features are basically noise and relevant but redundant features are nuisance [6]; we are not concerned with these features here [6]).

Typically the ligand’s chemical structure is represented by an *L*-dimensional vector $\mathbf{x} = (x_1, x_2,...,x_L)$. The elements $x_{l}$ ideally contain appropriate information about the ligand’s features, relevant for predicting its bioactivity. This bioactivity against a particular target or protein may be represented either numerically or as a class label; such classes (or class labels) are denoted henceforth by *k*, where *k = *1, 2, ..., *K* with *K* being the total number of classes of interest.

Identifying the relevant features **x** without errors is generally impossible. Usually both **x** and *k* are treated as random variables such that for a given **x** we have a distribution $p(k|\mathbf{x})$—the so-called class posterior probability—on the different possible classes [1, 7]. In practice, $p(k|\mathbf{x})$ that can assign a new ligand represented by **x** to the class minimising the probability of misclassification is induced from given prototype samples (a training dataset) [8, 9].

In Bayesian probabilistic settings, it is usually computationally easier to estimate $p(k|\mathbf{x})$ in terms of class probability (*p*(*k*)), evidence ($p(\mathbf{x})$) and class-conditional probability density function ($p(\mathbf{x}|k)$):

1 $$\begin{aligned} p(k|\mathbf{x})=p(\mathbf{x}|k)\times \frac{p(k)}{p(\mathbf{x})}, \end{aligned}$$

In cheminformatics, the main task of estimating $p(k|\mathbf{x})$ often reduces to inducing $p(\mathbf{x}|k)$ from the training dataset.

## Commentary

It is common practice nowadays to assume that the *L* relevant chemical structure features of the ligand can be encoded as a binary “vector” of 1’s and 0’s denoting presence (1) and absence (0) of these features—i.e., $x_l \in \{0, 1\}$ [10]. In practice, state-of-the-art feature selection techniques [3, 5] that are based on information theory are used to quantify the level of association between the features and the bioactivity. These techniques are also capable of quantifying the class-conditional interdependency among the features. However, in the light of the insightful work of Li on the peculiar but useful characteristics of the conditional dependence between two binary random variables [11], one might be able to go one step further; identify the $L^{\prime }$ features in the *L* relevant features whose relationship with the bioactivity is statistically significant, but whose class-conditional interdependency is statistically insignificant—i.e., retain features that are statistically non-redundant (and for that matter ignore or discard statistically redundant features).

In our probabilistic setting, $L^{\prime }$ relevant descriptors $\mathbf{x}^{\prime } = (x^{\prime }_1,x^{\prime }_2,...,x^{\prime }_L)$ being non-redundant entails that $p(\mathbf{x}^{\prime }|k)$ can be expressed as a product of $L^{\prime }$ class-conditional univariate probability density functions $p(x^{\prime }_{l}|k)$, i.e., $p(\mathbf{x}^{\prime }|k) = \Pi ^{L^{\prime }}_{l=1}p(x^{\prime }_{l}|k)$. This means that $p(k|\mathbf{x}^{\prime })$, which is what we are interested in estimating, can be given as

2 $$\begin{aligned} p(k|\mathbf{x}^{\prime }) = \Pi ^{L^{\prime }}_{l=1}p(x^{\prime }_{l}|k)\times \frac{p(k)}{p(\mathbf{x}^{\prime })}, \end{aligned}$$

Since $x^{\prime }_{l}\in \{0,1\}$, the univariate distributions $p(x^{\prime }_{l}|k)$ are Bernoulli [8, 12, 13], i.e. $p(x^{\prime }_{l}|k) = p(x^{\prime }_{l}=1|k)^{x^{\prime }_{l}}[1-p(x^{\prime }_l=1|k)]^{(1-x_{l})}$. In terms of these Bernoulli distributions, Eq. 2 modifies to

3 $$\begin{aligned} p(k|\mathbf{x}^{\prime })&= \Pi ^{L^{\prime }}_{l} p(x^{\prime }_{l}=1|k)^{x^{\prime }_{l}}[1-p(x^{\prime }_l=1|k)]^{(1-x_{l})}\nonumber \\&\quad \times \frac{p(k)}{p(\mathbf{x}^{\prime })} \end{aligned}$$

which can be further rewritten in an equivalent but more convenient form (see Chapter 4 of ref [8]):

4 $$\begin{aligned} g_{k}(\mathbf{x}^{\prime }) = \sum ^{L^{\prime }}_{l}x^{\prime }_{l}c_{kl} + d_k, \end{aligned}$$

where $c_{kl} = \log {\frac{p(x^{\prime }_{l}|k)}{1-p(x^{\prime }_{l}|k)}}$; $d_k = \sum ^{L^{\prime }}_{l=1}\log {(1-p(x^{\prime }_{l}|k))}+\log {\frac{p(k)}{p(\mathbf{x}^{\prime })}}$. Clearly, the discriminant function $g_{k}(\mathbf{x^{\prime }})$ is linear in **x**$^{\prime }$ [8, 12, 13]—irrespective of the nature of the association between the chemical structure of the ligand and its bioactivity. This is the consequence of the ligand’s relevant but non-redundant features being represented by a binary “vector”.

However, the situation can be different if non-redundant real-valued features are utilised to represent the chemical structure of the ligand. In this scenario the $L^{\prime }$ class-conditional univariate distributions $p(x^{\prime }_{l}|k)$ are not necessarily Bernoulli. Here $p(x^{\prime}_l|k)$ can be expressed in Hermite polynomial basis functions $\phi _{n}(x^{\prime}_{l})$ in variable $x^{\prime}_{l}$

5 $$\begin{aligned} p(x_{l}^\prime|k) = \sum ^{\infty }_{n}\alpha ^{k}_{nl} \phi ^{k}_{n}(x^{\prime }_{l}), \end{aligned}$$

where $\alpha ^{k}_{nl}$ are the appropriate coefficient values. Note that the *k* in $\alpha ^{k}_{nl}$ and $\phi ^{k}_{n}$ is just an index (not a power). Inserting Eq. 5 into Eq. 2 and then taking the logarithm of the resultant equation yields the following discriminant function

6 $$\begin{aligned} h_{k}(\mathbf{x}^{\prime }) = \sum ^{L^{\prime }}_{l}\Big [\sum ^{\infty }_{n}\alpha ^{k}_{nl}\phi ^{k}_{n}(x^{\prime }_{l})\Big ]+b_k, \end{aligned}$$

where $b_k = \log {\frac{p(k)}{p(\mathbf{x}^{\prime })}}$. Clearly $h_{k}(\mathbf{x}^{\prime })$ is not necessarily linear in **x**$^{\prime }$ even though the $L^{\prime }$ features utilised are class-conditionally independent [13]. Thus, for real-valued features, the resulting classifier is capable of representing a non-linear structure-activity relationship.

## Conclusions

In this commentary it has been noted that, when ligand features are represented by a string of binary numbers, one must end up with a linear model for describing the dependency (if any) between the chemical structure of a ligand and its bioactivity of interest—albeit in a classification setting. Such a linear model may be severely biased and limited in its predictivity. It was also pointed out that, where relevant real-valued features are used, the resulting model can be unbiased as it can adequately capture both linear and non-linear structure-activity relationships.

## References

[1] Mussa HY, Marcus D, Mitchell JBO, Glen RC (2015). Verifying the fully “Laplacianized” Naive Bayes and more. J Cheminform.

[2] Afzal AM, Mussa HY, Turner RE, Bender A, Glen RC (2015). A multi-label approach to target prediction taking ligand promiscuity into account. J Cheminform.

[3] Tourassia GD, Frederick ED, Markey MK, Floyd CE (2001). Application of the mutual information criterion for feature selection in computer-aided diagnosis. Med Phys.

[4] Battiti R (1994). Using mutual information for selecting features in supervised neural net learning. IEEE Trans Neur Nets.

[5] Peng H, Long F, Ding C (2005). Feature selection based on mutual information: criteria of max-dependency, max-relevance, and min-redundancy. IEEE Trans Patt Anal Mach Intel.

[6] Langley P, Sage S (1994) Induction of selective Bayesian classifiers. In: UAI94 Proceedings of the Tenth International Conference on Uncertainty in Artificial Intelligence, San Francisco, pp 399–406

[7] Mussa HY, Afzal AM, Mitchell JBO (2015). Parzen Window approach reduced to two vectors and one matrix. Pat Recogn Lett.

[8] Duda RO (1973) Pattern classification and scene analysis, 1st edn. John Wiley & Sons Ltd, New York

[9] Young TY, Calvert TW (1974). Classification, estimation, and pattern recognition.

[10] Willett P (2011). Chemoinformatics: a history. Comput Mol Sci.

[11] Li W (1990). Mutual information function versus correlation functions. J Stat Phys.

[12] Bahadur R R: A representation of the joint distribution of the responses to n dichotomous items. In *Studies in Item Analysis and Prediction*; ed. Salomon H: Standford University Press: Standford, CA, 1961

[13] Hand DJ, Yu K (2001). Idiot’s Bayes-not ao stupid after all?. Int Stat Rev.

